# Myeloid cells as IFNα producers in systemic lupus erythematosus

**DOI:** 10.3389/fimmu.2025.1562221

**Published:** 2025-07-17

**Authors:** Taiga Kuga, Asako Chiba, Goh Murayama, Sachiko Miyake

**Affiliations:** ^1^ Department of Immunology, Juntendo University Faculty of Medicine, Bunkyo-ku, Tokyo, Japan; ^2^ Department of Internal Medicine and Rheumatology, Juntendo University Faculty of Medicine, Bunkyo-ku, Tokyo, Japan

**Keywords:** SLE, pDC, monocyte, interferon, TLR, cGAS-STING, cellular senescence

## Abstract

Type I interferons (IFNs) play crucial roles in the pathogenesis of systemic lupus erythematosus (SLE). Plasmacytoid dendritic cells (pDCs) stimulated by Toll-like receptor (TLR) pathways have been thought to be the major producers of IFNα in patients with SLE. However, the responsiveness of pDCs from SLE patients to stimuli that produce IFNα differs depending on the type of TLR pathway involved. In addition to pDCs, monocytes from SLE patients were found to produce IFNα when responding to the cGAS-STING pathway. Here, we outline the major pathways that induce IFNα production by myeloid cells in SLE, and the possible mechanisms by which IFNα overproduction occurs by these cells. Finally, we discuss the current and future therapeutic strategies to regulate IFNα production in patients with SLE.

## Introduction

The involvement of type I interferons (IFNs) in the pathogenesis of systemic lupus erythematosus (SLE) is well established, and recent advances in the treatment of SLE with anifrolumab, a fully human anti-IFN α/β receptor antibody, have highlighted the importance of controlling the disease by inhibiting the type I IFN pathway. The treatment of patients with SLE with anifrolumab improved skin rash, arthritis, overall disease activity, and their health-related quality of life (1–4). Myeloid cells produce type I IFNs including IFNα when nucleic acid receptor pathways are activated. In this review, we describe the pathways and possible mechanisms by which IFNα production occurs in SLE.

## Nucleic acid receptor pathways

Type I IFNs, inflammatory cytokines that play a critical role in the self-defense system, are produced when nucleic acid receptors recognize microbial and viral nucleic acids (5, 6). During infection, epithelial cells and fibroblasts at the site of infection produce type I IFNs such as IFNβ, and then plasmacytoid dendritic cells (pDCs) produce large amounts of type I IFNs, particularly IFNα (7, 8). In addition, other myeloid cells such as monocytes, conventional dendritic cells, and macrophages also produce type I IFNs (7, 8). Previous reports suggested the activation of nucleic acid receptor pathways is associated with the overproduction of type I IFNs and the pathogenesis of SLE. In addition to exogenous nucleic acids, self-derived nucleic acids also induce the production of type I IFNs. In SLE, nucleic acids derived from apoptotic cells, those contained in neutrophil extracellular traps (NETs) and mitochondrial DNA released due to mitochondrial stress have been demonstrated to activate nucleic acid receptor pathways (9–11). Immune complexes composed of autoantibodies and nucleic acids have also been shown to induce IFNα by pDCs (12–15). Impaired DNA clearance also leads to the activation of nucleic acid receptor pathways and the production of type I IFNs in SLE. Genetic deficiencies of nucleic acid degradation molecules, such as extracellular DNase-I and TREX1, the most abundant 3’→5’ DNA exonuclease in cells, are associated with a lupus-like syndrome in mice and humans (16–18). Most patients with SLE have reduced DNase I activity (19, 20), which might be explained by anti-DNase antibodies being present in approximately 60% of sera samples from SLE patients (21, 22).

Among the Toll-like receptors (TLRs), TLR4 recognizes pathogen-associated molecular patterns on the cell membrane surface and TLR3/7/8/9 located in endosomes or lysosomes recognize nucleic acids. Upon ligand binding, TLR7/9 induce type I IFN production via the myeloid differentiation factor 88 (MyD88)/TRAF6/IRF7 complex, and TLR3/4 induce type I IFN production by activating interferon regulatory factor 3 (IRF3) through the Toll/IL-1R domain-containing adaptor inducing interferon-β factor (TRIF) pathway in a MyD88-independent manner (8, 23). IFNα production by pDC upon stimulation with immune complexes composed of autoantibodies and nucleic acids was suppressed by blocking FcgRII, and thus these immune complexes appear to be internalized into endosomes where they activate TLR7 and TLR9 pathways (24). pDCs constitutively express high levels of IRF7 and produce large amounts of type I IFNs via TLR7/8/9 (25). Overactivation of the TLR7 pathway caused autoantibody production and lupus-like disease in mice (26–30). *TLR7* polymorphisms have been shown to be associated with SLE (31–33). For example, a *TLR7* gain-of-function genetic variation (34) and overactive TLR7 pathway as a result of variants in *UNC93B1*, which binds TLR7 and is essential for TLR7 trafficking to the endosome (35, 36), have provided direct evidence that genetic variants related to TLR7 activation trigger human SLE pathogenesis. Recently, *UNC93B1* variants were reported in SLE patients (37, 38), and mice expressing a variant of *UNC93B1* developed spontaneous lupus-like disease (37).

Nucleic acid receptors such as cGAS (cGMP-AMP synthase) and the RIG-I-like receptor (RLR) family and melanoma differentiation-associated gene (MDA5) recognize viral nucleic acids and self-derived DNA in the cytoplasm. Upon the recognition of cytoplasmic double-stranded DNA, cGAS synthesizes cyclic GMP-AMP (cGAMP), a second messenger that binds to the transmembrane protein stimulator of interferon gene (STING) located in the endoplasmic reticulum. STING is then transported to the Golgi apparatus where it induces type I IFN production via the TBK1-IRF3 pathway (39). Several lines of evidence suggest the cGAS-STING pathway is activated in SLE. A mutation in *TMEM177*, which encodes STING, has been associated with SLE-like diseases (40, 41). The cGAS-STING pathway is typically activated in response to DNA derived from pathogenic microbes or viruses, but excess self-DNA, derived from apoptosis-derived membrane vesicles (42), neutrophil extracellular traps (11), and mitochondrial DNA (43) may also contribute to aberrant cGAS-STING activation in SLE. Indeed, cGAS expression is increased in peripheral blood mononuclear cells (PBMCs) from SLE patients (44). Because IFNα impairs mitochondrial metabolism and autophagic degradation leading to the accumulation of mitochondrial DNA in the cytosol, IFNα overproduction in SLE may also contribute to the activation of the cGAS-STING pathway (43). Autoimmune diseases in *Trex-1* and *DNase II*-deficient mice were shown to be dependent on the cGAS-STING pathway (45, 46), and other murine studies have demonstrated the contribution of the cGAS-STING pathway to enhancing type I IFN responses in lupus or lupus-like mouse models (47–49). These findings suggest that activation of the cGAS-STING pathway is deleterious in the pathogenesis of SLE by the induction of type I IFNs. However, cGAS or STING deficiency resulted in exaggerated disease in the MRL/lpr mouse model and pristane-induced lupus model (50). Further studies are needed to understand how the cGAS-STING pathway is involved in the pathogenesis of lupus.

## IFNα overproducing myeloid cells in SLE

**Figure 1:**
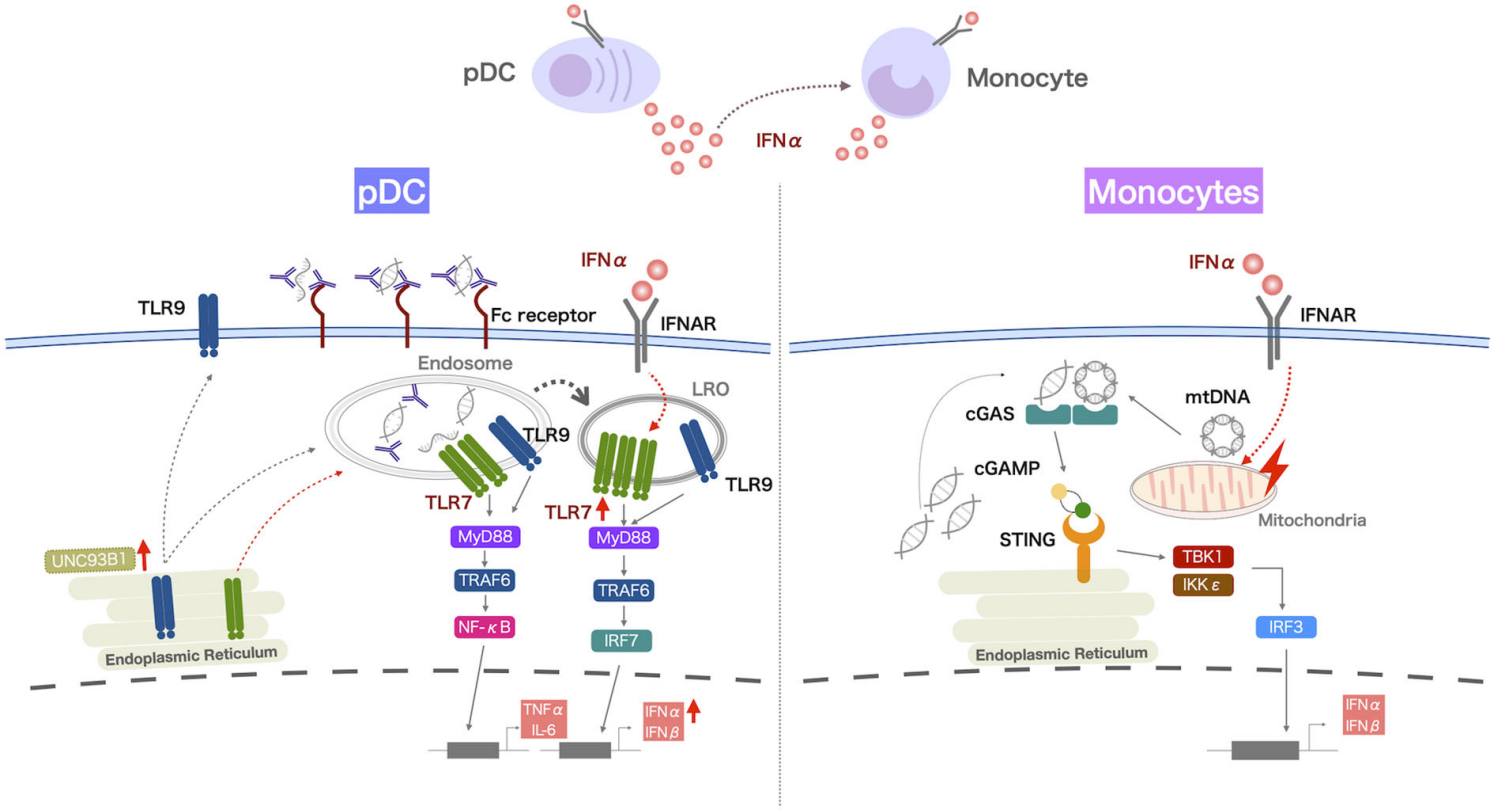
IFNα overproduction by myeloid cells in patients with SLE. IFNα production by pDCs from SLE patients is increased when they are activated via the TLR7 pathway, and the increased retention of TLR7 in lysosome-related organelles (LRO) of pDCs may contribute to this enhanced response. Monocytes from SLE patients produce increased levels of IFNα after activation of the cGAS-STING pathway. IFNα production by pDCs and monocytes correlates with disease activity in SLE, and increased TLR7 responsiveness is observed in pDCs, even in SLE patients with stable disease. These TLR7 and cGAS-STING pathways are enhanced by exposure to IFNα. Thus, IFNα production by pDCs may underlie the pathology of SLE and increased IFNα production by pDCs and monocytes due to the increased nucleic acid load contributes to disease activity.

pDCs are known to be the most potent IFN-I producing cells and thus have been widely proposed to be the dominant source of type I IFN production in SLE. The early ablation of pDCs in BXSB lupus-prone mice prior to disease onset ameliorated lupus nephritis, reduced the tissue expression of IFN-induced genes, and diminished the cascade of IFN-mediated responses, including the reduction of antinuclear antibodies, splenomegaly, and abnormal expansion of T and B cells (51). In humans, the administration of a monoclonal antibody against blood DC antigen 2(BDCA2) expressed on pDCs decreased expression of IFN response genes in the blood, and reduced disease activity of skin disease and arthritis in SLE, suggesting that pDCs may indeed be an important source of type I IFNs in SLE (52–54). The TLR7 and TLR9 pathways induce IFNα production by pDCs. However, pDCs from SLE patients had increased IFNα production when stimulated with a TLR7 agonist, but reduced IFNα production in these cells when stimulated with a TLR9 agonist (**Figure 1**) (55, 56). Given that most nucleated cells can produce IFN-I during viral infection (57), it is important to consider IFN-I production by other cell subsets. In our study focusing on the cGAS-STING pathway, numbers of IFNα-producing cells among PBMCs from SLE patients were increased when stimulated with 2’3’-cGAMP, a STING-activating ligand (58). IFNα production by pDCs and conventional DCs was also increased in patients with SLE, but the primary IFNα-producing cells among PBMCs were monocytes. In addition, monocytes from SLE patients had higher STING expression and IFN-I expression upon 2’3’-cGAMP stimulation compared with those from healthy controls (**Figure 1**) (58, 59).

Increased IFNα production by pDCs and monocytes was positively correlated with disease activity in SLE (55, 56, 58), suggesting that these cells may contribute to the pathogenesis of SLE. Interestingly, the capacity to produce IFNα by pDCs was also increased in patients with stable disease (55). In patients with cutaneous lupus erythematosus, the type I IFN signature was enhanced in keratinocytes from lesional and nonlesional skin, and pDCs were the major subset among myeloid cells in nonlesional skin (60). Thus, IFNα production by pDCs may underlie the pathogenesis of SLE and lupus-like disease. When the cGAS-STING pathway is activated by the accumulation of DNA in the cytosol, monocytes produce IFNα, which can cause disease flares of SLE. Because monocytes are migratory cells, they may produce IFNα at the sites of inflammation. The single-cell RNA-seq analysis of kidney samples from patients with lupus nephritis revealed that non-classical monocytes migrated to the kidney and differentiated into monocyte-derived macrophages, with their characteristics shifting from inflammatory to phagocytic (61). In lupus models, Ly6C^lo^ monocytes, considered non-classical monocytes, were reported to infiltrate into the kidneys of MRL/lpr mice and ABIN1 (Tnip1)-deficient mice (62), as well as the central nervous system of NZB/NZW and FcgRIIB^-/-^ *Yaa* mice (63). In imiquimod (IMQ)-induced lupus mice, Ly6C^hi^ monocytes were increased in the lymph nodes and Ly6C^lo^ monocytes expressing high levels of TLR7, adhesion molecules, and cytokines such as IL-6, were increased in the peripheral blood and infiltrated into the kidneys (64). Ly6C^hi^ and Ly6C^lo^ monocytes upregulated the expression of proinflammatory cytokine genes when stimulated with a TLR7 agonist, and only Ly6C^hi^ monocytes upregulated IFNα genes when stimulated via the cGAS-STING pathway. It is unknown whether human IFNα-producing monocytes are CD16-negative classical monocytes because activated monocytes downregulate CD16 expression. Studies in mice suggested that Ly6C^hi^ classical monocytes with IFNα-producing capacity migrated to the lymph nodes, whereas Ly6C^lo^ non-classical monocytes infiltrated into the site of inflammation (62, 64). IFN-responsive genes were upregulated in microglia from FcgRIIB^-/-^ *Yaa* mice and Ly6C^lo^ monocytes present in the central nervous system of FcgRIIB^-/-^ *Yaa* mice may produce IFNα (63). Pseudotime analysis of the RNA sequencing data of myeloid cells in the blood and skin lesions from patients with cutaneous lupus erythematosus indicated the transition of circulating non-classical monocytes to skin infiltrating CD16^+^ DCs, which was associated with the expression of type I IFN (60). Thus, circulating monocytes may also become IFNα-producing cells and contribute to disease pathogenesis at the site of inflammation.

## Mechanisms of IFNα overproduction by myeloid cells in SLE

IFNα production by pDCs was increased when activated by a TLR7 agonist, and decreased by the TLR9 pathway, but the expression levels of TLR7 and TLR9 were reported to be comparable in pDCs from SLE patients and healthy controls (65). IRF7 activation for type I IFN expression requires TLR localization to lysosomes, and UNC93B1 is important for the trafficking of TLR7 and TLR9. Of note, we observed the increased retention of TLR7 in the lysosomes of pDCs from SLE patients (55). TLR9 competes with TLR7 for trafficking by UNC93B1 (66), and the accelerated localization of TLR7 to lysosomes may suppress TLR9 trafficking by UNC93B1. Exposure to IFNα increased IFNα production by pDCs and this was associated with increased TLR7 localization to lysosome-related organelles (55). Thus, the *in vivo* exposure of pDCs to IFNα in SLE may enhance their responses to TLR7 stimulation. UNC93B1 variants were shown to cause hyperresponsiveness to TLR7 stimulation, but not to TLR9 stimulation (37, 38). Thus, UNC93B1 variants may also contribute to the increased IFNα production by pDCs stimulated by a TLR7 agonist.

We found that STING expression and its colocalization with TBK1 was increased in monocytes from SLE patients (58). In contrast, monocytes from healthy individuals produced low levels of IFNα upon cGAS-STING activation, although their *in vitro* exposure to IFNα induced the expression of STING and IFNα (58). IFNα-induced STAT1, a transcription factor known to be activated by cytokines including IFNα, binds directly to the promoter of STING to enhance STING induction (67), and exposure to IFNα induced the colocalization of STING and TBK1 (58), which might be associated with the effect of IFNα on the induction of mitochondrial DNA accumulation in the cytoplasm. Thus, prior exposure to IFNα may accelerate the TLR7 and cGAS-STING pathways. Increased STING expression was accompanied by increased phosphorylated-mTOR? in SLE monocytes, suggesting that increased STING expression is also related to impaired autophagy. Indeed, the treatment of monocytes with rapamycin, an inhibitor of mTOR, decreased STING and IFNα production by SLE monocytes. Thus, impaired autophagy may also contribute to the increased STING and IFNα expression by SLE monocytes (58).

**Figure 2:**
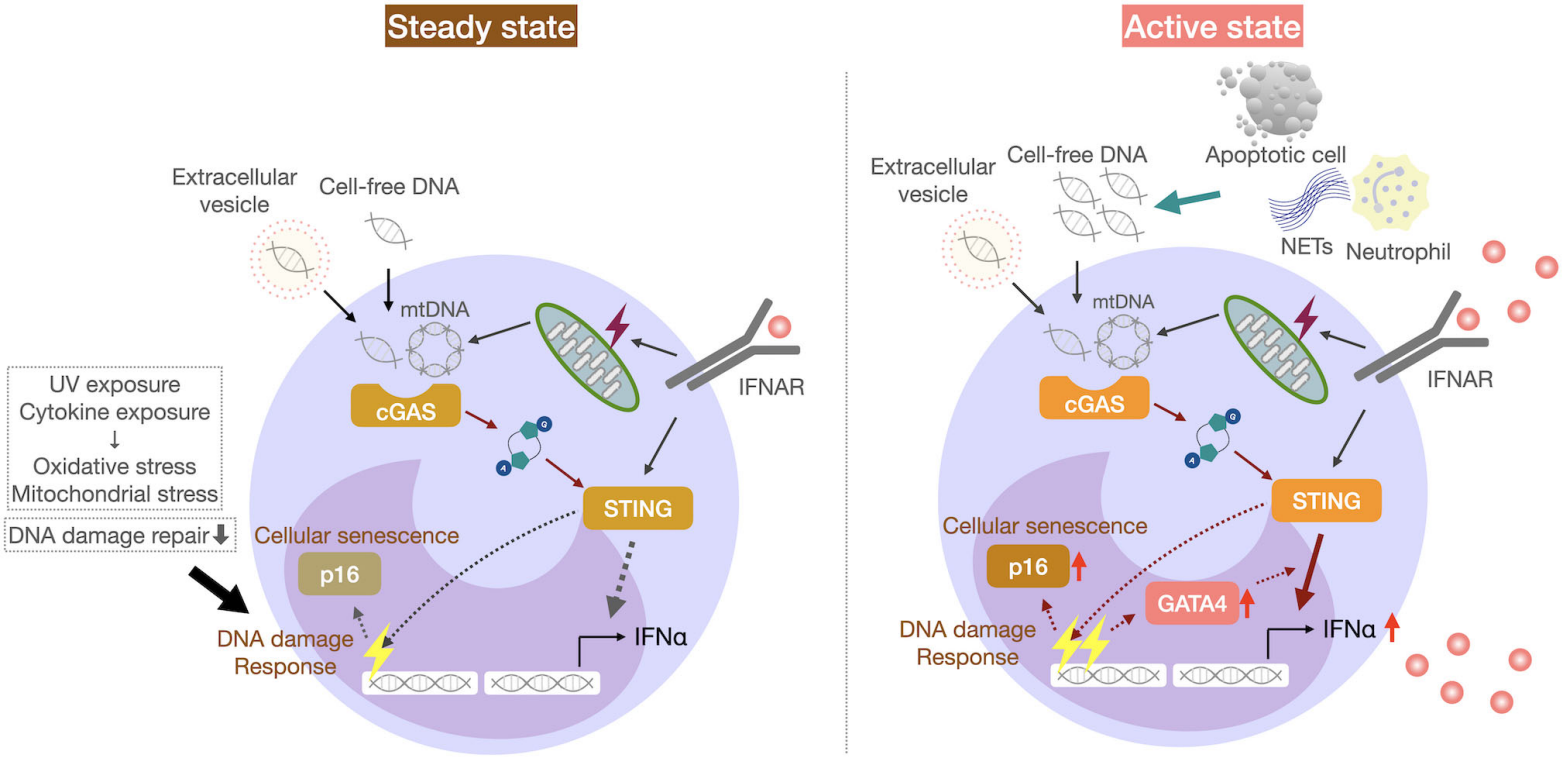
IFNα overproduction by senescent monocytes in patients with SLE. SLE monocytes from SLE patients exhibit cellular senescence-like features, including p16 expression and increased senescence-associated β-galactosidase activity. GATA4 expression increases the responsiveness of the cGAS-STING pathway and IFNα production by monocytes, which may increase the disease activity in SLE.

To uncover the cell-intrinsic mechanisms underlying enhanced IFNα production by STING stimulation, we performed the transcriptomic analysis of monocytes from SLE patients and *in vitro* IFNα-exposed monocytes from healthy individuals, and found that the transcription factor GATA4 was upregulated in monocytes from SLE patients (**Figure 2**) (68). GATA4 is a key transcription factor that contributes to the production of cytokines including IL-1 and IL-6, via NF-κB in cells with cellular senescence, a phenomenon known as the senescence-associated secretory phenotype (69). Cellular senescence refers to a phenomenon whereby cells stop proliferating due to various stresses and factors that cause DNA damage. GATA4 expression also enhanced IFNα production in monocytic U937 cells (68). GATA4 binds to the enhancer region of the IFIT family. Furthermore, IFIT3 was reported to be upregulated in SLE monocytes, and its inhibition reduced type I IFN production by monocytes activated by the cGAS-STING pathway (59). In addition to GATA4 expression, SLE monocytes exhibited cellular senescence-like features, including high CDKN2A expression and increased senescence-associated β-galactosidase activity (68). The DNA damage response, as measured by γ-H2AX levels, was reported to be increased in monocytes (70), B cells (71), and bone marrow-derived mesenchymal stem cells from SLE patients (72). The *in vitro* exposure of activated human B cells to IFNα and human carcinoma cell lines to a mixture of IL-1β, IFNγ, and TNFα has been shown to induce DNA damage responses in these cells (71, 73). Thus, IFNα overproduction in SLE may contribute to enhanced DNA damage responses (71). Thus, the accumulation of cytoplasmic DNA, such as micronuclei, due to DNA damage induces cellular senescence and may also activate the cGAS-STING pathway.

## Therapeutic strategies for SLE by inhibiting IFNα production

Some of the current therapeutic strategies for SLE have been shown to suppress IFNα production. Many lines of research have clearly demonstrated that hydroxychloroquine (HCQ) reduces disease flares in SLE patients (74, 75) by inhibiting the activation of endosomal TLRs, particularly TLR7 and TLR9 (76, 77) and antigen presentation (78), as well as reducing NETs formation (79). Indeed, HCQ suppressed IFNα production by pDCs exposed to CpG-A (80). pDCs isolated from cutaneous lupus erythematosus and SLE patients administered HCQ had significantly lower IFNα production upon TLR7 or TLR9 stimulation compared with patients not receiving HCQ (80, 81). Furthermore, HCQ levels in the blood of patients with cutaneous lupus erythematosus correlated negatively with the IFNα-producing capacity of their pDCs upon TLR9 stimulation, with a weaker correlation observed upon TLR7/8 stimulation (80). HCQ suppresses the formation of cGAMP from cGAS; therefore, HCQ may also inhibit IFNα production via the cGAS-STING pathway (82).

Prior exposure to IFNα increased IFNα production by pDC and monocytes (55, 56, 58). Thus, anifrolumab treatment may reduce IFNα production by these cells. The MUSE study, a trial of anifrolumab, showed that SLE patients with high IFN gene signature (IFNGS) or high disease activity had decreased numbers of lymphocytes, neutrophils, pDCs and monocytes, and the decrease in these cells was reversed after anifrolumab treatment (83). This anifrolumab treatment effect was shown to be associated with the suppression of interferon-inducible chemokines such as interferon gamma-induced protein 10 (IP-10) and IFN-inducible T cell alpha chemoattractant (ITAC). Therefore, anifrolumab treatment may reduce the migration of immune cells, including those capable of producing IFNα, to sites of inflammation.

Plasma dsDNA from SLE patients was shown to activate the cGAS-STING pathway in monocytic cells when using a cell-based reporter system that detected the bioavailability and inducing activity of IFN-I (42, 84). Type I IFN bioavailability was decreased in the plasma of SLE patients treated with double filtration plasmapheresis (DFPP) and this was associated with decreased plasma cDNA levels (84). This suggests that the beneficial effects of DFPP may be related to the removal of nucleic acids in addition to autoantibodies.

Rapamycin, a drug that inhibits T-cell proliferation, has shown efficacy in a phase 1/2 clinical trial for the treatment of SLE (85). Autophagy was suppressed in SLE monocytes, and rapamycin reduced STING expression and IFNα production by these cells, suggesting that the cGAS-STING pathway may be a therapeutic target for the suppression of IFNα production. Indeed, several inhibitors of cGAS or STING are already in development (86). In SLE patients, the source of IFNα may vary between individuals, and TLR7 and pDCs might also be a good therapeutic target. Inhibitors of TLR7/8 such as E6742, afimeoran (NCT04493541), and enpatoran (NCT05540327) are being or have been investigated in clinical trials for SLE. E6742, a selective dual antagonist for TLR7/8, has been investigated in a double-blind phase 1/2 study in SLE, and 57.1% of patients achieved a positive response on the British Isles Lupus Assessment Group-based Composite Lupus Assessment (BICLA), compared with 33.3% in the placebo group (87). SLE patients with active cutaneous lupus erythematosus (CLE) treated with anti-BDCA2 antibody litifilimab (also known as BIIB059) showed an approximately 50% reduction in IFNGS expression in whole blood 24 hours after treatment, and litifilimab treatment was highly effective in normalizing IFN response proteins (myxovirus resistance protein 1 and IFN-induced transmembrane protein 3) in lesional skin and reducing CD45+ immune cell infiltration (52). Phase 2 studies of litifinimab demonstrated the reduction of Cutaneous Lupus Erythematosus Disease Area and Severity Index Activity scores and in the number of swollen and tender joints in SLE (53, 54). The efficacy of litifilimab is being further investigated in phase 3 trials (NCT04895241 and NCT04961567). Therapeutic strategies targeting specific immune cells or nucleic acid receptor pathways may be an option depending on which cell type is the prominent IFNα producing cell in SLE.

## Conclusions

Although increased IFNα production by pDCs was observed in SLE patients including those with stable disease, IFNα production by monocytes in SLE patients positively correlated with disease activity. Thus, IFNα overproduction by pDCs may enhance the responsiveness of pDCs and monocytes to nucleic acid receptor pathways, and IFNα overproduction by monocytes may be responsible for the disease activity in SLE. Thus, the suppression of the TLR pathway may be more important for the maintenance of stable disease and the activation of the cGAS-STING pathway may need to be suppressed to inhibit the disease flares in SLE. The future therapeutic targets of nucleic acid receptor pathways and myeloid cells should be decided depending on the disease status in SLE.
